# Validating FR20 and D‐FR20 for Bromodomain and Extra‐Terminal Domain Inhibitor Resistance in Triple‐Negative Breast Cancer

**DOI:** 10.1002/advs.78443

**Published:** 2026-10-25

**Authors:** Man Sun, Dan Zang, Jun Chen

**Affiliations:** ^1^ Department of Oncology The Second Hospital of Dalian Medical University Dalian City Liaoning Province China

To the Editor,

We read with interest the study by Liu et al. [1], which combines time‐series single‐cell RNA sequencing, perturbational transcriptomics, and functional experiments to examine resistance to bromodomain and extra‐terminal domain inhibitors in triple‐negative breast cancer. The single‐cell design resolves resistant‐state heterogeneity, the GPX4 experiments support a functional role for ferroptosis suppression, and testing of filgotinib provides an experimental link between computational screening and re‐sensitization. These contributions also raise a broader question: when should a resistance signature be regarded as a translational tool? We define the translational threshold as evidence that a model has a prespecified and calibrated output, reproducible performance in independent patient‐derived and treatment‐annotated cohorts, added value beyond established clinical variables, and a decision rule linked to an actionable time point. For drug prioritization, this threshold also requires stable candidate ranking, comparison with alternative methods, mechanism‐specific validation, and evidence that the selected combination improves activity beyond either monotherapy. Against these criteria, FR20 and D‐FR20 are promising discovery frameworks, but their current evidence does not yet support clinical deployment.

FR20 is constructed by summing normalized expression of 20 ferroptosis regulators selected from FerrDb V2, with a weight of +1 for suppressors upregulated in resistant cells and −1 for drivers downregulated in resistant cells. Its biological basis, directional association with induced JQ1 resistance, and replication across datasets support its interpretation as a resistance signature. The score is transparent, computable at single‐cell resolution, and applicable to both single‐cell and bulk transcriptomic data. However, all nine validation cohorts are based on cell‐line systems. Cell‐level comparisons do not replace independent biological replication, the 22RV1 and LNCaP analyses rely on group means because of limited replicates, and the association in the Cancer Cell Line Encyclopedia is borderline. The reported pan‐cancer area under the dose‐response curve analysis stratifies cell lines from multiple tumor types by the upper and lower quartiles of FR20 and then compares JQ1 response, with a higher area indicating lower sensitivity, rather than testing a prespecified threshold in patients with triple‐negative breast cancer. Clinical development would therefore require a locked formula and cutoff, platform and batch robustness, external validation in patient‐derived models and treatment‐annotated cohorts, and reporting of discrimination, calibration, and incremental clinical value [2]. These steps would determine whether FR20 measures a reproducible biological state or can guide a treatment decision.

D‐FR20 extends this signature through 720 216 small‐molecule perturbation instances from the Library of Integrated Network‐based Cellular Signatures. For each perturbation, gene set enrichment analysis estimates normalized enrichment scores for the upregulated ferroptosis suppressors and downregulated ferroptosis drivers. A weighted connectivity score combines the two components when their directions are opposite, and the most negative scores define the leading candidates. This mechanism‐anchored and scalable design is a clear strength, and filgotinib was supported by viability, half‐maximal inhibitory concentration, colony formation, and zebrafish xenograft experiments. Nevertheless, signature reversal does not establish that a compound is the optimal partner for JQ1. Rankings may depend on cell context, dose, exposure time, and the chosen connectivity metric. Only one leading candidate was functionally advanced, and the study did not test rank fidelity across top‐ranked and lower‐ranked compounds or quantify pharmacologic interaction. Prior work has benchmarked connectivity methods against curated drug relationships using partial area under the receiver operating characteristic curve at low false‐positive rates and has also examined differences among connectivity scores [3, 4]. A similar evaluation of D‐FR20, followed by blinded testing of high‐, intermediate‐, and low‐ranked candidates, dose‐matrix analyses using established synergy metrics, and GPX4‐dependent rescue experiments across several triple‐negative breast cancer models, would support its use as a prioritization framework. Current data show that D‐FR20 can nominate a plausible re‐sensitizer; they do not yet establish calibrated ranking of combination therapies.

The time‐series design also orders transcriptional states from dimethyl sulfoxide exposure through JQ1 treatment, resistance, and retreatment, but Monocle2 and CytoTRACE infer transcriptional continuity rather than clonal history. The observed trajectory could therefore reflect treatment‐induced state transition, selective expansion of rare pre‐existing tolerant cells, or both. Single‐cell studies of triple‐negative breast cancer have shown that sensitive and resistant clones can coexist before or during treatment, supporting the need to separate state change from clonal selection [5]. This distinction has direct clinical consequences. If FR20 identifies baseline tolerance, pretreatment measurement could support patient enrichment or evaluation of an upfront combination. If it reflects treatment‐emergent adaptation, serial sampling would be more informative and combination therapy could be introduced when the score rises. Applying an upfront combination without this distinction could expose patients without a tolerant state to unnecessary toxicity. Lineage tracing, clonal barcoding, and longitudinal sampling linked to drug response are therefore required to define when FR20 should be measured and acted upon.

Liu et al. provide a valuable link between single‐cell analysis, ferroptosis biology, and experimental drug testing. The next step is to align each framework with its intended use. FR20 requires context‐matched clinical validation and a prespecified decision threshold; D‐FR20 requires comparative ranking and combination‐specific validation. Resolving whether the measured state is present before treatment or emerges during therapy will then determine whether these tools are best suited to baseline stratification, longitudinal monitoring, or the timing of combination treatment.

## Funding

This work was supported by the National Natural Science Foundation of China (Grant No. 82203056).

## Conflicts of Interest

The authors declare no conflicts of interest.

## Data Availability

Data sharing not applicable to this article as no datasets were generated or analysed during the current study.
